# An Australian brain bank and the future of alcohol and major neuropsychiatric disorders research

**DOI:** 10.3389/fneur.2026.1746346

**Published:** 2026-02-20

**Authors:** Julia Stevens, Caine C. Smith, Dhiraj Maskey, Mario Novelli, Jennifer Bronfenbrener, Markus J. Hofer, Greg T. Sutherland

**Affiliations:** 1 New South Wales Brain Tissue Research Centre, Charles Perkins Centre and School of Medical Sciences, Faculty of Medicine and Health, The University of Sydney, Sydney, NSW, Australia; 2 School of Life and Environmental Sciences and Charles Perkins Centre, Faculty of Science, The University of Sydney, Sydney, NSW, Australia

**Keywords:** alcohol use disorder, biobanking, neuropathology, neuroscience, post-mortem brain

## Abstract

Post-mortem human brain banks are a key resource for researching brain diseases. The New South Wales Brain Tissue Resource Centre (BTRC) is a brain bank that uniquely focuses on the recruitment, preparation, and dissemination of tissue from patients with alcohol use disorder and controls. Our controls are prospectively followed through their lifetime via a self-reported questionnaire and yearly updates, and these standardised data allow future matching to disease cases based on individual study needs. Brain banks are expensive to run, and their sustainability is an ever-present topic of concern worldwide. In this review, we explore how the BTRC is adapting to a changing research environment by updates to our banking pipeline, before considering different models whereby brain banks can add greater value to research of the brain and other organs. First, brain tissue research is undergoing a major transformation with the rapid uptake of single-cell and spatial platforms. Brain banks must ensure that their protocols are optimised and updated to match the requirements of these new platforms. The BTRC has moved to rapid fixation of tissue and is trialling freezing protocols that minimise cytoarchitectural damage. Second, post-mortem brain banks are inherently retrospective and cannot ordinarily contribute to research during a donor’s lifetime. However, brain banks can also expand their portfolio to include clinical samples and derivatives such as cell lines, and this may promote greater donor interest in subsequent brain donation. Third, brain banks have traditionally run as stand-alone operations given their unique reliance on invasive autopsies and whole organ banking. However, with the increased interest in brain–body interactions, multi-organ tissue banks holding both clinical and post-mortem samples could enable the discovery of general disease mechanisms. Finally, the single-cell and spatial platforms are producing data at a phenomenal rate. Rather than seeing data derived from tissue disseminated to disparate repositories, banks could curate the data in-house and enable dry-lab research alongside their traditional focus on tissue studies. Overall, post-mortem brain banking is an important part of the brain research environment, but the banking pipeline must be designed to maximise benefits for donors and future generations.

## Introduction

Brain diseases are often uniquely human and difficult to model, and this has slowed the discovery of new therapeutic and preventative avenues. Human post-mortem brain banking is a crucial contributor to medical research investigating the pathogenesis of changes occurring directly in the human brain. As rates of mental illness, neuropsychiatric and neurological disorders rise globally (1), there is an increasing need for well-characterised, high-quality, systematically biobanked central nervous system (CNS) tissues.

The majority of brain banks worldwide are affiliated with tertiary referral clinics, and their collections reflect research interest in high-profile disorders such as Alzheimer’s, Parkinson’s, and motor neuron diseases. In this study, brain banks serve a dual role of diagnostic confirmation through neuropathological examination and tissue dissemination (2).

In contrast, very few brain banks concentrate on mental health disorders such as addiction, rare disorders including childhood dementias, and controls. The NSW Brain Tissue Resource Centre (BTRC) has been operating at the University of Sydney, Australia, for well over 25 years. During this time, the BTRC has aimed to bridge the gaps in human tissue for research with a collection focused on individuals with neuropsychiatric disorders, including alcohol use disorder (AUD), and neurologically ‘normal’ controls (3). Currently funded by the National Institute on Alcohol Abuse and Alcoholism (NIAAA), the BTRC is specifically dedicated to the collection, storage, and distribution of brain tissue for AUD researchers globally. The BTRC currently has a collection of 522 whole brains from largely Caucasian (White) donors and 20% female, including 116 donors who meet the clinical criteria for alcohol use disorder.

There are many challenges associated with post-mortem brain banking, with public awareness, clinical support, protocol refinement, and funding vital to the long-term sustainability of every brain bank (4). There are additional challenges when banking for stigmatised disorders or rare diseases, specifically around recruitment and establishing sufficiently large collections.

One of the major issues is the regulatory environment and the lack of specific rules for brain banking (5). For example, in Australia, brain banking is covered by a ‘patchwork’ of intersecting federal and state-based legislation. These are primarily Human Tissue Acts but may also include Coroners and Privacy Acts. Privacy provisions are particularly complex because of the additional oversight by Institutional Review Boards (IRBs). The latter have standards set by the governing body of medical research in Australia, the National Health and Medical Research Council (NHMRC). However, formal national guidelines only relate to organ donation for transplantation rather than research (6), whilst their more informal ‘information paper’ for biobanking has no specific reference to brain banking (7). The situation in Australia is also very dynamic with the Federal Privacy Act and the NSW Human Tissue Act currently under review.

There is no doubt that post-mortem brain tissue research is important and likely to be more so with the discovery potential of the latest spatial multi-omic tools (2). However, banks also need to continually assess how they can add further value to medical research and the lives of patients and their families. In this study, we describe how the BTRC has approached these issues and how we continually review and, if necessary, modify our protocols to ensure that our tissues meet the expectations of our researchers using the latest molecular tools. Moreover, we describe our transition away from a classic banking model with no direct benefit to our donors during their lifetime to one where we also facilitate research through clinical research support, clinical sample banking, and multi-omic data hosting—a modern brain biolibrary (8).

### The post-mortem brain banking pipeline

**Figure 1:**
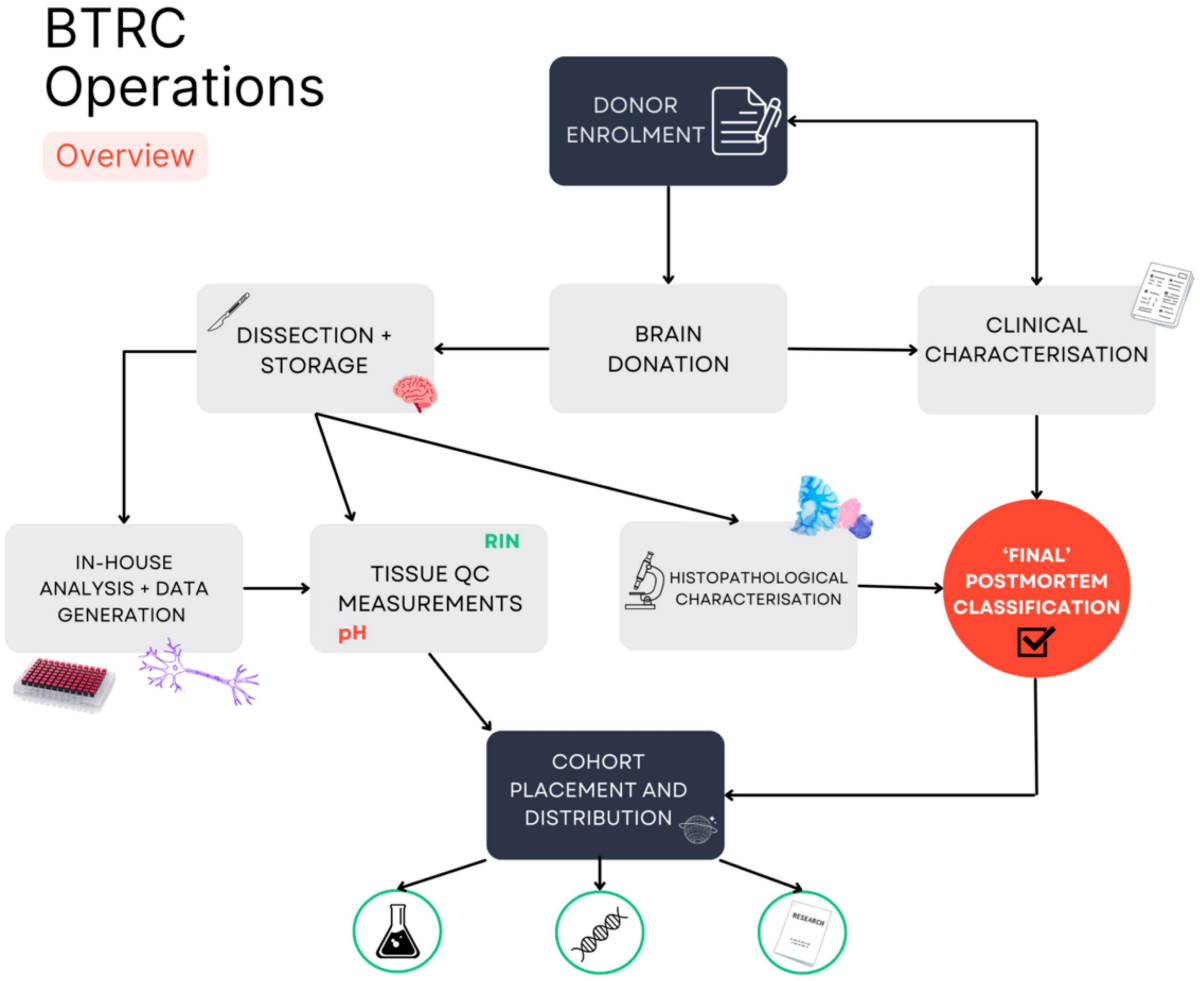
Overview of Brain Tissue Resource Centre operations. A schematic shows the key steps in the recruitment, retrieval, and dissemination of post-mortem brain tissue for at the BTRC. Neurologically normal or affected persons are made aware of the Using our Brains (UoB) prospective donor program by their clinician or advertising. The BTRC sends interested persons a participant information pack and if agreeable consents them along with their next of kin. At death, a brain is removed at a forensic mortuary and transferred to the BTRC. Self-reported medical information along with retrospectively examined medical records are used to create a clinical diagnosis. At the BTRC, the brain is split—half is frozen and half fixed by rapid perfusion. Small frozen tissue samples are used for measuring brain pH and RNA Integrity Number (RIN), with DNA extracted for genotyping. A set of fixed standard blocks are embedded and sectioned and stained are for neuropathological examination. Once a pathological diagnosis is confirmed, the cases are placed in diseased or control cohorts.

The BTRC has developed a unique brain banking model to recruit individuals affected by AUD along with neurologically normal controls (Figure 1).

#### Recruitment

The main pathway for brain donation to the BTRC is currently through the prospective brain donor programme, Using our Brains (UoB). Members of the community are invited to donate their brain, spinal cord, and a liver sample by enrolling with the UoB during their lifetime. The UoB requires the consent of both the participant and their senior next of kin (SNoK). Upon sign-up, donors are required to provide longitudinal lifestyle and medical information through a comprehensive questionnaire and then update it via an annual survey. Given that ‘Using our Brains’ controls are used for studies across the spectrum of neuropsychological diseases, our protocols and survey questions for living donors are as generic as possible. Our surveys also include standardised alcohol drinking behaviour questions to allow clinical criteria to be established [e.g., Alcohol Use Disorder Identification Test-Consumption (AUDIT-C) (9)]. After death, medical records are obtained from donors’ medical practitioners and specialists to determine clinical classifications. In Australia, only persons over 18 years of age can consent to tissue donation. The BTRC does not recruit minors (<18 years). The programme has been well-received, reflecting our recent findings that Australians are highly supportive of brain donation (10).

More generally, community perceptions of brain donation have been mixed across surveyed nations and communities. General awareness of brain donation for research is certainly low, both in Australia and globally (10–12). Considering this, we are now actively advertising the UoB via social media, alcohol advocacy groups, and individuals attending our partner drug and alcohol treatment clinics. The latter relationship has been difficult to manage given the adage that 10% of those with alcohol use disorder seek treatment, although this may be as low as 8%, and then only 10% of those remain in treatment (13). Drug and alcohol specialists therefore have a difficult decision to judge whether a patient may be amenable to a conversation around future brain donation and the timing of that conversation.

Brain donation into the BTRC has also occurred through a long-standing partnership with the state forensic service and coroner, who work in concert to handle coronial cases in NSW. This involves retrospective consenting via SNoK of donors with a presumed history of alcohol misuse who have not died under suspicious circumstances. This is followed by retrieval of medical records as described above. However, declining triple-cavity autopsy rates in the forensic setting mean less opportunity for obtaining bankable tissue (14–16).

Biobanking of non-CNS organs is relatively easy via collection during routine biopsy or surgical organ removal, and typically banking can be run in parallel and is complementary to diagnostic pathology services. CNS organs, however, are only available post-mortem. Outside the coronial system, adult autopsies in hospitals in Australia are also extremely uncommon (17). It is vital that brain banks commit time and resources to publicly promote the benefits of brain donation to policymakers and other relevant organisational bodies. It is also likely that brain banks will soon need to use their own institutional facilities, such as anatomy morgues and trained staff, to enable rapid brain retrieval. Rapid retrieval where the post-mortem interval (PMI) is well below 24 h is an industry standard where the antemortem state of macromolecules is largely maintained and downstream applications, including cell line generation, are facilitated (18, 19). This will increase expenses and require additional operations support, but these costs can be dispersed amongst other potential users of rapidly retrieved post-mortem tissue.

#### Tissue processing

The BTRC is notified of donor death through our on-call pager system, which is always monitored. Organ retrieval is carried out by trained technicians at New South Wales Health Pathology Forensic Services before further dissection by the BTRC in a dedicated brain suite at our neuropathology laboratory housed at the University of Sydney. Brains are hemisected, with one hemisphere and contralateral hindbrain fixed in 15% neutral-buffered formalin. The remaining half is frozen and stored at −80 °C. Further information detailing developments in tissue processing is outlined below. The spinal cord and liver are also processed and stored as fixed and frozen samples. Standard blocks are taken and processed as formalin-fixed paraffin-embedded (FFPE) blocks or as smaller frozen blocks to enable diagnostic staining and ease of access to subregions that are regularly requested for research (Table 1). Each dissected case is documented photographically as the whole brain and tissue slices, and volumetrics of the whole brain and fixed hemisphere are measured. A full neuropathological examination is performed by a certified neuropathologist for each case to determine a diagnosis.

**Table 1:** BTRC’s standard formalin-fixed paraffin-embedded (FFPE) and fresh frozen blocks.

Region	Brodmann area	FFPE block	Fresh frozen block	H&E	Garvey	Abeta	AT8	Masson’s trichrome stain
Superior frontal cortex	9	Yes	Yes					
Dorsolateral prefrontal cortex	46							
Posterior cingulate	23		Yes					
Choroid plexus			Yes					
Pineal gland		Fixed only	Yes					
Meninges			Yes					
Olfactory bulb		Fixed only	Yes					
Circle of Willis		Fixed only						
Prefrontal cortex	6	Yes	Yes	Yes		Yes		
Hippocampus		Yes	Yes	Yes	Historical	Yes	Yes	
Striatum		Yes	Yes	yes		Yes (if necessary)		
Amygdala		Yes	Yes	Yes				
Anterior cingulate cortex	24	Yes	Yes	Yes				
Midbrain		Yes	Yes	Yes		Yes (if necessary)		
Thalamus		Yes	Yes	Yes				
Superior temporal gyrus	22	Yes	Yes	Yes	Historical	Yes	Yes	
Inferior temporal gyrus		Yes	Yes	Yes	Historical	Yes	Yes	
Supramarginal gyrus	40	Yes	Yes	Yes				
Primary visual cortex	17, 18	Yes	Yes	Yes				
Primary motor cortex	4	Yes	Yes	Yes		Yes	Yes	
Cerebellum		Yes	Yes	Yes				
Cervical spinal cord		Yes	Yes	Yes				
Vermis		Yes	Yes	Yes				
Upper thoracic spinal cord		Yes	Yes	Yes				
Lower thoracic spinal cord		Yes	Yes	Yes				
Lumbar spinal cord		Yes	Yes	Yes				
Sacral spinal cord		Yes	Yes	Yes				
Mammillary body/hypothalamus		Yes	Yes	Yes				
Pons		Yes	Yes	Yes				
Medulla		Yes	Yes	Yes				
Liver		Yes	Yes	Yes				Yes
BA5		Yes		Yes				
Spinal tag		Yes		Yes				
Globus pallidus/putamen		Yes		Yes				
Caudate/putamen/nucleus accumbens		Yes		Yes		Yes (if necessary)		

In addition to the diagnostic work-up of each case, the BTRC analyses the quality of the fresh frozen tissue by determining the RNA integrity number (RIN) and pH from a small sample of cerebellum. DNA is also extracted from frozen tissue for whole-genome genotyping as discussed below.

#### LIMS

Biobanking relies on effective tracking of samples and data. This requires a sophisticated but easy-to-use Laboratory Information Management System (LIMS), but these work most effectively and can be automated when samples are stored in a uniform manner. Given the varied combination of stored tissues, the BTRC has developed a bespoke in-house LIMS to manage donors, samples, case retirement when regions are expended, researcher details, and project data. This system has recently been extended to track how each case is utilised and the publications that arise from tissue use.

#### Tissue distribution

The BTRC is an open-access biobank. All researchers can apply for brain and spinal cord tissues for ethically approved projects. Enquiries are assessed for tissue availability before a completed application is peer-reviewed by an expert panel for significance, feasibility, and originality. Tissue request reviewers are researchers with previous experience in the use of post-mortem brain tissue. Alcohol-related applications are reviewed by the NIAAA-managed Scientific Advisory Board, whereas all applications for non-AUD projects are accepted if tissue supply is feasible and the project is approved by our independent scientific review committee. To date, 147, 000 fixed and frozen samples have been supplied across 685 projects. Tissue has been supplied as fresh frozen samples, fresh frozen sections, FFPE sections, fixed ‘floating’ sections, and fixed tissue blocks. The popularity of requests for tissue types has varied over the years as new technologies for downstream applications have emerged. At the start of the BTRC, FFPE sections were the most requested tissue for immunohistological and stereological studies. Interest in fresh frozen tissue then increased as molecular technologies in the omics field exploded. The appearance of spatial technologies has meant that FFPE sections have now come back into focus for users of post-mortem brain. Whilst fresh frozen tissue is superior in terms of RNA quality, the cytoarchitecture is often suboptimal in the human brain relative to FFPE tissue. FFPE sections with <24 h fixation provide a suitable alternative for spatial work with the percentage of fragments greater than 200 bp (DV200) at or greater than 60% despite RIN less than 2 (20).

### Changing research landscapes

Coordinated preservation and collection of human brains began in the late 19th century, with these practices evolving into the modern brain banks of today (21). Early post-mortem brain research was largely histological in nature but evolved into omics work, requiring high-quality fresh frozen tissues. This trend continues with the latest spatial platforms, particularly spatial omics, that require both high-quality molecular components and highly preserved cytoarchitecture. The ability to supply bank tissue that is fit for purpose across evolving technologies requires an acute awareness of researcher needs, in addition to flexibility, time, and proactive networking across international brain banks to create effective and standardised best practices. Ideally, the brain bank team or closely affiliated members should be active researchers who can predict trends, oversee tissue protocol development, and perform the beta-testing of new methodologies.

The spatial technologies on the market are varied (e.g., *MERFISH, NanoString Geo, and 10x Visium*) and growing rapidly. The intricacies inherent to these techniques and level of optimisation mean that banks and researchers may be better placed working collaboratively. One issue is that banks are tasked with prolonging tissue resources and ensuring that tissue is supplied in an equitable manner. It may be easier for banks to do these techniques on behalf of researchers as opposed to providing whole FFPE or frozen tissue blocks to researchers so these techniques can be firstly optimised and then used at the researcher’s discretion.

Another major issue with the spatial technologies is the trade-off between resolution and capture area. This increases the already large challenge of reproducibility between studies that aim to sample the exact same brain region. Currently, regions are visually identified and manually processed by the staff of each bank, leading to variation in the exact site sampled. A coordinate-based schema for directing dissection would be a major step forward in this regard, particularly if adopted widely. There are a number of initiatives in play to achieve this through three-dimensional (3D) reconstitution of 2D tissue slices (22), which often incorporate *in vivo* or *ex vivo* MRI (23–25). One of the first comprehensive attempts to create ‘cytoarchitectonically parcellated’ regions of interest, combining histological sections with *in vivo* MRI, was carried out in macaques (26). This utilised whole fixed brains, sectioned in celluloid moulds, Nissl-stained, and converted to grey-scale images for MRI registration via a multi-stage algorithm. As a labour-saving task, they repeated the registration, substituting photographic images of the sections taken before staining, and the results were similar. Given that the majority of banks hemi-sect a brain, photographic images of slices of both hemi-brains taken prior to storage may represent the most effective method of the 3D reconstruction (24). Gazula et al. (24) used surface scans (3D scanning) of the whole brain, rather than just relying on a probabilistic atlas. This method is agnostic to slice thickness and thus is suited to the hemi-brain approach, where the side to be frozen is sliced fresh, with variable thickness, relative to the more rigid, and thus finely sliceable, fixed hemi-brain.

### Moving forward

The BTRC has ethics approval from the Human Research Ethics Committee (equivalent to an IRB) at the University of Sydney, which covers all stakeholders, including in-kind contributors, to the consenting, retrieval, and dissemination of tissue. The UoB consent form has been developed as ‘broad consent’, such that each donor agrees to allowing tissue to be provided to researchers internationally for unspecified research and that data arising from this research may be shared in public repositories. This has been an important point, as many researchers use post-mortem brain tissue from the BTRC or other banks under a waiver from their own IRB. It is now mandated by many funding bodies and journals that omics data be uploaded to data repositories and that effective data management should operate under findable, accessible, interoperable, and reusable (FAIR) principles (27). However, these same IRBs are then called upon to guarantee to data repositories that the work producing these data had been carried out in an ethical manner. They are increasingly deferring to brain banks to ensure that the work is in line with the original consent of the donor. This is problematic as two sources of genomic data can be proven by a third party to come from the same individual and may in some jurisdictions amount to de-identification. These third parties will increasingly bring artificial intelligence-assisted technologies to data mining, but the corollary is that fewer donors may consider donation if their anonymity, privacy, and sense of data ownership cannot be guaranteed (28). This may also differ between those affected by a disease and healthy volunteers (29).

In response to researcher uptake of spatial technologies, which requires both high-quality RNA and cytoarchitecture, the BTRC has refined and continues to refine the processing of donor tissue. The fixed hemisphere is now perfused-fixed in 15% neutral-buffered formalin, followed by immersion for up to 24 h; a reduction from 3 weeks (diffusion fixation) as previously reported (3). We are currently exploring changes to our procedures to freeze tissue to enable better cytoarchitecture in this tissue. Initial trials of rapid freezing methods and varying cryoprotectants were carried out in sheep necropsy brain tissue (20), with trials of freezing methods for human tissue ongoing.

As research technologies evolve and change in popularity, so do brain regions of interest. Post-mortem human brain is not easily accessible and has limitations; therefore, animal models and imaging studies have long led the way into brain research. As new technologies have allowed deeper analysis of animal tissue and human images, various new regions of interest have emerged as candidates for validation in human tissue. To keep up with this, the BTRC continuously adds regions to be dissected as standard blocks (Table 1).

Given the relative scarcity of human post-mortem brain tissue available for research, the BTRC is committed to exploring ways to maximise research output using minimal tissue. One such option is the creation of tissue microarrays (TMAs). Briefly, FFPE TMA blocks for one case are created containing tissue cores from 10s to 100s of parent blocks for multiple brain regions (30). This allows concurrent staining and comparison of many brain regions in one section. Multiplex immunofluorescence (mIF) is another method that can maximise outcomes whilst reducing reagent and tissue use. Combining TMAs with mIF is a particularly effective way to broadly sample multiple brain regions simultaneously to look at the regional impact of neurodegenerative diseases (30) or AUD (53). Furthermore, mIF can be combined with spatial transcriptomics technologies to provide unprecedented data on the juxtaposition of pathology and cellular subtypes. We have shown how more effective mIF can be used with 24-h FFPE tissue, but many important markers used in neuroscience can be robustly used in 3 weeks—or longer—of fixation times. mIF is constantly evolving but remains relatively expensive when using commercial technologies that use covalent amplification reactions to circumvent antibody specificity and fluorophore abundance issues. At the same time, the BTRC has shown that more traditional methods can be used with iterative strip-incubate cycles to approach the outputs achieved with newer technologies (Maskey et al., in this issue). Finally, digital pathology is increasingly important in human post-mortem tissue analyses. The BTRC recognises the need to use machine learning algorithms to speed up the quantification of cells and pathology and their spatial interactions whilst maintaining stereological principles of sufficient sampling (31). Current progress at the BTRC is best described as semi-quantitative (30) with ongoing work to use newer deep learning algorithms such as *nnU-Net* (32) to achieve full automation.

### Deriving additional donor data from post-mortem assays

Historically, pH and RINs have been used as the standard measurements to assess macromolecule quality in brain samples. FFPE tissue would typically not be the first choice for RNA-based experiments. However, new sequencing-based spatial transcriptomics platforms are designed for FFPE tissue (33). In tissue fixed for 24 h, the integrity of RNA measured in terms of fragments exceeding 200 bp (DV200) can exceed 60%, even though the RIN is less than 2 (20), whereas standard blocks typically result in DV200 approximately 25% or lower. In comparison, frozen cryosections are typically approximately a RIN of 7, with DV200 of 80–90%, but, as discussed elsewhere, current freezing methods compromise cytoarchitecture and potentially allow transcripts to disseminate from their cell of origin.

The BTRC has typically recorded blood alcohol content (BAC) to confirm active drinking in coronally sourced AUD cases. Then using medical records, our team has estimated lifetime alcohol consumption, DSM IV and 5-based alcohol dependence, and AUDIT-C score for all donors. More recently, we have augmented our current drinking determinants by measuring phosphatidylethanol (PEth), a direct biomarker of alcohol use, in frozen brain tissue using a robust in-house liquid chromatogrpahy mass spectometry (LCMS) workflow (34). PEth is an effective indicator of alcohol use in the months prior to death and particularly useful when BAC is unavailable.

The BTRC has genotyped the majority of cases using the Axiom UK Biobank Genotype Array platform to further characterise and broaden the downstream analysis that can be performed using our tissue. This is a genome-wide platform with 805,000 single-nucleotide polymorphism, including common mutations known to result in disease (35, 36). Researchers can opt to receive whole-genome data, which allow for analyses such as GWAS to be performed, or QTL analysis when combined with existing transcriptomic or methylome data that have been generated using BTRC tissue (37). They also allow the BTRC to select a cohort for research based on common variants such as apolipoprotein ε2, ε3, and ε4.

### The next Frontiers

As discussed above, funders and an increasing number of journals are asking that data on which published findings are based be made available to other researchers. This not only meets the FAIR principles (data that is findable, accessible, interoperable, and reusable) but also aligns with the basic tenet of scientific endeavours as being reproducible (38). As methodologies get more complex, they are creating a so-called ‘data deluge’ for both clinicians and researchers (39). It is unclear whether raw data or normalised data should be uploaded, given the expert bioinformatics skills required to re-analyse the former. Brain banks are theoretically in a strong position to host research data derived from their tissue, as they can ensure donor and region fidelity and explore outliers by investigating medical records and potentially performing meta-analyses. The BTRC has facilitated several recent meta-analyses of transcriptomic data but has stopped short of hosting raw data (45–47). Other issues of concern include maintaining de-identification inherent to the donor consent. One option that is used by large epidemiological studies such as the UK Biobank is to set up an independent data enclave, where both data curation and analysis are carried out in a secure environment, with the bank ensuring that access and results made public meet consent and privacy stipulations. Such a data coordination centre would be another avenue where a brain bank can directly facilitate research and, importantly, make AUD data available to non-content experts who may derive hitherto unknown relationships with other diseases or general pathomechanisms.

The BTRC has been funded by the NIAAA in some form for 30 years to support AUD research. Focusing on a stigmatised disease such as AUD has many challenges, but this is offset to some extent by its commonality. An even more challenging focus is rare brain diseases, particularly in a geographically large country such as Australia. For diseases that typically have a prevalence of less than one per million, it is almost impossible for a single bank to collect a cohort of reasonable study power. One option is to make more use of the more than 60 brain banks around the world and the estimated ~100,000 banked brains (40). There are many obstacles to creating a single physical collection, such as the majority of banks not accepting tissue from outside their own country. One option that the BTRC is pursuing is a virtual international brain bank that curates tissue from rare brain disease cases worldwide. This solves a problem for researchers by establishing whether sufficient cases exist for their work, although it does not solve the likelihood of multiple tissue transfer agreements and courier fees. Moving forward, a virtual brain bank could act as a nidus for greater internationalisation in brain banking, where individual banks could be the custodians for a single rare disease collection, making dissemination of tissue to researchers simpler and cheaper.

### Brain and body bank

There is much interest in finding non-invasive, clinical brain biomarkers. Plasma-based biomarkers are well established for cardiovascular and liver disorders but in their infancy for brain disease. A brain bank could expand its collection to include DNA, plasma, and cell lines. There are already existing centralised repositories for DNA, serum, and peripheral blood mononuclear cells (PBMCs) for disorders such as Alzheimer’s disease (AD), although these have not been coordinated with the subsequent donation of brain tissue from the same individuals. Recently, one such facility expanded to incorporate a biomarker assay laboratory to run AD-specific assays such as Aβ 40, Aβ 42, and P-tau 217 but also more general assays of brain damage, including Glial Fibrillary Acidic Protein (GFAP) and Neurofilament Light (NFL) (48). Given the increasing certainty that plasma assays do reflect conditions behind the blood–brain barrier (41), the BTRC could expand its collection and monitor AUD patients for potential brain damage whilst also simultaneously providing serum and cell lines to researchers. This would allow the BTRC to facilitate AUD research whilst the patient is still alive and may even encourage subsequent brain donation. It has the added benefit of contributing to the clinical characterisation of future donors.

Expanding a post-mortem tissue bank to one housing clinically derived samples would require substantive philanthropic government support. In Australia, federal research infrastructure funding is given based on maximum beneficiaries. Therefore, brain banks should consider greater interoperability with the wider biobanking community. One option would be a multi-organ bank that stores blood and cells from potential donors within the patient’s lifetime as above but also sets the foundations for deriving hypothesised systemic manifestations of brain diseases, including addictions and vice versa (42). For example, the combination of a brain, heart, liver, and cancer tissue biorepository planned for the University of Sydney would be useful for the exploration of multi-organ dysfunction due to chronic alcohol consumption (49). There are also interesting connections between somatic mutations in cancer and germline mutations in neurodegenerative diseases (50), whilst brain-specific diseases such as multiple sclerosis may represent the CNS manifestation of past, systemic viral infection that may have also affected other organs (51).

A related idea is the rapid tissue retrieval of multiple organs from a post-mortem donor. Outside of brain banking, the majority of other organ researchers can rely on surgical biopsy tissue. The use of post-mortem tissue is less common, but then the donation of deceased tissue as a legacy option is largely unknown to Australians (10). A multi-organ, multi-disease focus would allow banks to make a much more compelling case for national infrastructure funds. Finally, donated tissue is only as good as the clinical information held on that individual individual. There are currently studies, such as the UK Biobank, that are longitudinally creating the most characterised individuals in research history. There needs to be more effort to bank tissues from these individuals. In NSW, the 45 and Up study has a similar focus, and some biobanking of participants has been carried out (52). A multi-organ tissue bank from these individuals could create a unique legacy for medical research.

### Research outcomes

Measuring sample utilisation and the outcomes arising from tissue use is vital in assessing the impact and value of the collection (43). Researchers are required to report publication and presentation outcomes to the BTRC annually. More recently, the BTRC has used an effective method for tracking and identifying publications where tissue or BTRC-derived datasets have been utilised. Briefly, a robust set of search terms was devised to run an active search of citation databases, Dimensions and Scopus, to alert the BTRC when a relevant publication is released. Since 2001, 776 papers, including preprints and papers published using a BTRC-derived dataset, have been published in peer-reviewed journals, with over 1,000 oral and poster presentations given at conferences. Further analysis of these publications has revealed 456 individual authors from 443 different institutions across 44 countries. The collection has 47,961 lifetime citations and a mean relative citation ratio of 2.03.

### Internationalisation of brain banking

The regulatory challenges faced in Australia are both like and different from those across national borders (5). However, there are many reasons why the internationalisation of (post-mortem) brain banks would be useful (4, 40). Chiefly amongst them is that this small industry could punch further above its weight in terms of research outputs if protocols and data collection were standardised worldwide. Danner et al. described this as a more ‘collective initiative’ to meet researchers demands (4). One stretch objective for the industry is to centralise sufficiently sized (powered) collections to facilitate research into uncommon or rare brain diseases. The transfer of tissue, data, and funds across state or country borders are all current impediments to such initiatives, and many countries are grappling with the same general privacy issues as Australia. A useful starting place would be an international virtual brain bank or database that curates and catalogues all the brains available worldwide, e.g., Brain Bank Connect (44). This could easily morph into an international community of practice in brain banking, setting the bar for best practice, and potentially hosting data, but importantly providing a precedent for bankers to advocate locally for greater internationalisation of their tissue and associated data.

## Conclusion

**Figure 2:**
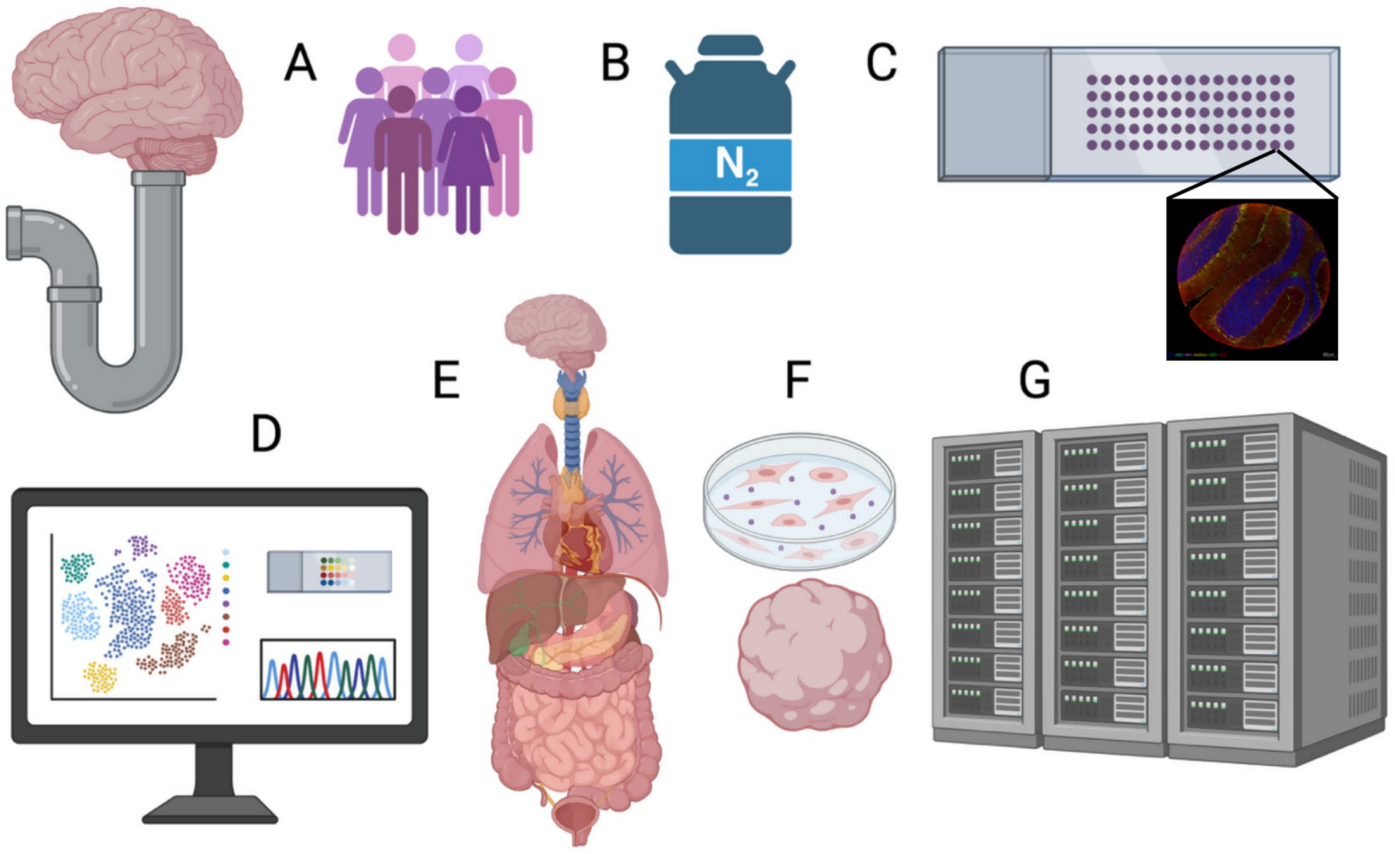
Modernising the human post-mortem brain bank pipeline. A schema shows how BTRC are modifying the brain banking pipeline to add greater value to human post mortem issue including: **(A)** Wider prospective recruitment using partnerships with advocacy groups; Rapid tissue retrieval with modification. **(B)** Frozen and FFPE issue preparation. **(C)** Tissue microarrays in combination with multiplex immunofluorescence imaging (mIF). **(D)** Using mIF in combination with single cell and spatial platforms. There are options for brain banks to become more utilitarian including **(E)** inter-operability with other organ bank or curating blood or skin samples from prospective donors such as that can be converted into **(F)** cell lines and organoids. **(G)** Finally, a modern brain bank could host omics data derived from their issue in an independent data coordination centre.

The provision of post-mortem brain tissue for research has traditionally relied on in-kind support from hospital neuropathology departments, coronial/forensic pathology facilities, and tertiary research clinics to provide tissue to researchers. These relationships partly dictate the disease focus but more so funding opportunities. Rarely, brain banks have achieved financial independence, with most relying on sporadic funds, including project grants associated with or affiliated with researchers or, on occasion, infrastructure support. Infrastructure funding will compete with other forms of biobanking, or, as in Australia, compete for infrastructure funding across the breadth of scientific enquiry. Project funding will favour the high-profile diseases, making the goal of resourcing rare or stigmatised disease research extremely difficult. The BTRC has taken a multi-faceted approach to these challenges (Figure 2). First and foremost, we have modified our protocols to be compatible with the latest spatial technologies that are currently revolutionising brain biology. Second, we have used various channels and established partnerships to publicise the idea of brain banking as a legacy for individuals or families. Third, we have worked closely with addiction medicine specialists to re-imagine the consenting process for individuals with alcohol use disorder. Moving forward, we intend to expand the remit from a purely physical biobank with limited capacity to supporting rare disease research through online databases, directly facilitating research by hosting a data enclave for multi-omic data derived from our tissues, exploring blood-based biomarkers to monitor AUD in the clinic, and partnering with other tissue biobanks to exploit potential synergies in brain and body disease mechanisms. These solutions can be summarised as creating a brain bio-library that solves problems for and gives value to multiple stakeholders and, in so doing, gives post-mortem brain banking the best chance of achieving long-term sustainability (8).
